# Deep Brain Stimulation Programming 2.0: Future Perspectives for Target Identification and Adaptive Closed Loop Stimulation

**DOI:** 10.3389/fneur.2019.00314

**Published:** 2019-04-03

**Authors:** Franz Hell, Carla Palleis, Jan H. Mehrkens, Thomas Koeglsperger, Kai Bötzel

**Affiliations:** ^1^Department of Neurology, Ludwig Maximilians University, Munich, Germany; ^2^Graduate School of Systemic Neurosciences, Ludwig Maximilians University, Munich, Germany; ^3^Department of Translational Neurodegeneration, German Center for Neurodegenerative Diseases (DZNE), Munich, Germany; ^4^Department of Neurosurgery, Ludwig Maximilians University, Munich, Germany

**Keywords:** deep brain stimulation, machine learning, adaptive, feedback, DBS target, reinforcement learning

## Abstract

Deep brain stimulation has developed into an established treatment for movement disorders and is being actively investigated for numerous other neurological as well as psychiatric disorders. An accurate electrode placement in the target area and the effective programming of DBS devices are considered the most important factors for the individual outcome. Recent research in humans highlights the relevance of widespread networks connected to specific DBS targets. Improving the targeting of anatomical and functional networks involved in the generation of pathological neural activity will improve the clinical DBS effect and limit side-effects. Here, we offer a comprehensive overview over the latest research on target structures and targeting strategies in DBS. In addition, we provide a detailed synopsis of novel technologies that will support DBS programming and parameter selection in the future, with a particular focus on closed-loop stimulation and associated biofeedback signals.

## Introduction

Deep brain stimulation (DBS) has become the treatment of choice for movement disorder, such as Parkinson's disease (PD), medically intractable essential tremor (ET) and complicated segmental and generalized dystonia (1). In addition, DBS is increasingly used in other neurological disorders like neuropathic pain and epilepsy, and is being investigated for psychiatric disorders (2), such as obsessive-compulsive disorder, depression and Tourette syndrome and neurodegenerative diseases like Alzheimer's disease (3). DBS is thought to modulate the function of the target region by applying electrical current to the area (4). Recent reviews propose that DBS likely acts through multimodal, non-exclusive mechanisms including immediate neuromodulatory effects on local and network-wide electrical and neurochemical properties, synaptic plasticity and long-term neuronal reorganization, potentially also providing neuroprotective effects and leading to neurogenesis (4–7).

DBS surgery involves implantation of electrodes into one of several target regions and administering electrical current pulses that are generated by an implanted impulse generator. Although the effects of DBS on for example Parkinsonian symptoms and quality of life are generally satisfying (8), the clinical outcome may vary between patients (9) and side effects can be induced (10) due to the stimulation of different functional pathways or structures nearby the original target. New approaches, such as current steering (11) are able to restrict the volume of tissue activated (VTA) (12) and therefore promise a more precise stimulation of neural structures. Improving the initial targeting and later stimulation of specific neural structures and pathways involved in the generation of pathological neural activity as well as avoiding others will be a crucial point for improving the clinical DBS effect and, at the same time, limiting side-effects.

The setting of DBS parameters to optimize therapy is time-consuming and will likely get more complicated with new technological developments, introducing an ever increasing combination of parameters like pulse duration, stimulation frequency, stimulation contacts and so forth. In open loop DBS, which is the current standard protocol, these stimulation parameters are set by a clinician in a trial and error procedure and remain constant until manually updated, irrespective of disease fluctuations. In a closed loop DBS system, a sensor continuously records a feedback signal, a so-called biomarker, which is ideally correlated or causally linked to a clinical symptom. A second major point of interest in DBS research therefore is to develop more sophisticated strategies and automated algorithms on how to program and adjust stimulation parameters in a precise and effective manner.

## Target Structures

Contemporary research in humans features investigations into different network structures connected to individual DBS targets and explores structural networks (13, 14) involved in the generation of disease symptoms. There are currently a handful of FDA approved DBS targets, including the subthalamic nucleus (STN), the internal segment of the globus pallidus (GPi), the nucleus ventralis intermedius (ViM), as well as several other investigational targets used for, often more than one for a given disorder or symptom (15). A popular target for DBS in medically intractable tremor, like Parkinsonian or essential tremor is the ViM. Studies using tractography show structural connectivity between ViM and motor cortical, subcortical, brainstem and cerebellar sites (16). Various other research groups show that the dentato-rubro-thalamic tract in the subthalamic region is implicated in tremor control (17) and report successful guidance of DBS surgery based on fiber tracking (18). Comparing STN DBS near tremor frequency in PD and DBS of the ventrolateral thalamus in ET, Cagnan and colleagues describe differences in the response of the behavioral tremor characteristics. They reason that different networks could be involved in essential and Parkinsonian rest tremor and conclude that these differences will be important in developing future strategies for closed loop DBS for tremor control (19).

Studies in dystonia patients have shown that ventral GPi stimulation is more efficient in alleviating dystonic symptoms (20). Using diffusion tensor tractography for investigating the connectivity patterns of different target structures and DBS electrode locations, Rozanski et al. report substantial differences in connectivity of dorsal and ventral GPi. The authors interpret their results in favor of functional differences in the ventral and dorsal GPi and recommend that specific targeting could play an important role in promoting distinct effects of DBS (21).

While PD patients show similar improvement in motor function after GPi- and STN-DBS (22), STN DBS is superior in improving off-drug phase motor symptoms (23). Therefore, the STN is often the preferred target to treat Parkinsonian symptoms, such as bradykinesia, tremor and rigidity. Accola et al. used STN LFP recordings from PD patients to investigate the relation between subthalamic fiber connectivity and oscillatory activity. The dorso-lateral portion of the STN, which shows the highest beta power in the STN, predominantly projected to premotor, motor, but also to associative and limbic areas. Ventral areas are connected to medial temporal regions, like hippocampus and amygdala (13). Recently, Tinkhauser et al. reported that beta oscillations recorded from directional contacts can be used as a predictor of the clinically most efficient contacts for stimulation in patients with PD (24). Various research groups (25–30) suggest that the posterior dorsolateral subthalamic region next to the red nucleus could be a “sweet spot” to help guiding DBS electrode placement in PD. However, the small size of the STN and its proximity to different axonal projections (31) can result in multiple side effects during high-frequency stimulation.

In summary, these results highlight the relevance of targeting specific (sub)-structures and networks in improving the clinical outcome after DBS surgery.

### Improving Surgical Planning, Evaluation, and Stimulation

Functional neurosurgery has been driven by technological innovations and DBS has evolved over the years, including new approaches to surgical targeting, evaluation and in the delivery of therapy at the target. For a detailed overview see Gross and McDougal (32). Improving and personalizing the targeting of specific (sub)-structures and avoiding others will be crucial for improving the clinical effect and limiting stimulation induced side-effects. New evolving technologies are turning away from classical cylindrical electrodes toward directional stimulating leads. The VANTAGE study, a multi-center study investigating the benefits of using segmented electrodes and multiple-source axially asymmetric directional DBS could show that such an approach leads to similar therapeutic effects as the standard approach without steering. A follow up study reports that axially asymmetric current can reduce adverse effects as well as efficacy thresholds in a highly individual manner, while also expanding the therapeutic window as compared to ring-mode DBS (33).

New software now allows for a patient-specific reconstruction of DBS leads based on MRI and post-operative CT imaging, the reconstruction of nuclei and fiber tracts adjacent to stimulation sites and the mapping of intra- and perioperative electrophysiological recordings (34, 35). For instance, Lead-DBS, now available in version 2.0, is a semi-automated toolbox to model deep brain stimulation electrode locations based on structural and neurophysiological imaging (34, 36). This toolbox now contains PaCER, a fully automated tool for electrode trajectory and contact reconstruction (37). Lauro et al. provide the open source software systems DBSproc and DBStar for clinical research which co-register CT and MR data for individual target localization and diffusion tractographic analysis from automatically detected DBS contacts (38, 39). On the industry side, Boston Scientific bought Cicerone DBS (40), a platform for stereotactic neurosurgical planning, recording, and visualization for DBS initially developed by the McIntyre lab and turned it into the commercial available software GUIDE. Medtronic initially offered comparable software called Optivize and recently replaced it with its sequel, SureTune 3. The company Brainlab, which has recently partnered with Boston Scientific to develop GUIDE XT, also offers a DBS surgery planning software called ELEMENTS which enables displaying target structures, fiber tracts as well as electrode trajectories.

The VTA is a concept to model the spatial dimension of stimulation for a given set of stimulation parameters (12, 41–43). It can be calculated from individual therapeutic impedance and stimulation energy (total electrical energy delivered, TEED) (44). With a 3D brain atlas and MRI data, the VTA can provide an approximate reconstruction of brain structures surrounding the DBS electrode as a 3D activate/non-activate image. A clinical application of the prediction of the spatial extent of VTA was reported to be helpful in optimizing DBS parameter settings in PD patients (45). However, the application of VTA remains limited due to a lack of impedance calculation in the model and differential strength-duration curves of the response of axons with different diameters because VTAs are derived from volume conductor models with a homogenous and isotropic tissue medium and the axonal trajectories are assumed to be perfectly straight and perpendicular to the electrode shaft, as for example in DBSproc and DBStar (38, 39).

As the electric fields generated during multi-contact stimulation become more complex, new approaches are needed minimize the prediction error for VTA and to quantify axonal and pathway responses in patients-specific models (46, 47). The clinical software StimVision provides another algorithm to calculate the VTA using the artificial neural network technique to facilitate tractography-based DBS targeting (48). Tractography is a modeling technique used to visually represent nerve tracts in 3D space using data collected by diffusion MRI (49). Results from tractography can be combined with post-operative computational modeling to determine the VTA based on electrode contacts, as the implanted electrodes can influence activity not only in gray matter structures but might also influence activity in surrounding white matter structures, thereby potentially influencing networks (50–52). The influence of fiber pathways in DBS has been shown with blood flow, glucose metabolism and blood oxygenation level dependence (BOLD) imaging techniques in multiple studies (53–55), supporting the hypothesis that DBS affects larger neuronal networks with subsequent downstream axonal activation. Sweet and colleagues combined results from tractography with post-operative computational modeling in patients with tremor-dominant PD identifying that the most efficient VTA stimulates the dentatothalamic fiber tract. As mentioned above, this tract probably plays an important role in the occurrence of tremor in PD and targeting it may alleviate tremor symptoms (43).

Advancements in imaging methods, such as ultra-high field MRI and new learning algorithms (34, 56–59) promise to refine our conception and understanding of different neural structures and their wiring in health and disease and will support the investigation of personalized target structures, thus possibly individualizing DBS surgery.

## New Sensing Devices and Feedback Signals

Today, DBS systems stimulate in an open-loop manner, meaning that stimulation parameters are pre-programmed and are not responsive to changes in the patient's clinical symptoms or in the underlying physiological activity. Although open-loop stimulation is state of the art, limitations like overall efficiency, reduction of efficiency over time or side-effects have become more obvious with growing clinical experience. DBS therapy adjustment also remains time-consuming, requiring clinicians to evaluate numerous combinations of stimulation parameters in order to achieve the optimal outcome. Selecting the right combination among many possibilities can have a major impact on the therapeutic effect (60). DBS practice currently requires patients to follow-up for months post-operatively to optimize the clinical effect of DBS. Disease and patient specific biomarkers could ideally help optimize therapy and help finding the right DBS parameter.

Medtronic now offers the implantable and rechargeable neurostimulator Medtronic® ACTIVA RC + S, a research system following the Activa PC + S system, which records electrophysiological signals from the implanted DBS electrodes and also offers inertial measurements. New miniature implants (61) with names like Neural dust (62) or Neurograins (63) will push the boundary of signal collection even further and ultimately promise to provide read and stimulation capabilities with a far greater spatial and temporal detail than available at present. There now are several companies actively pursuing brain computer interface technology by developing new neural implants, ranging from traditional medical device manufacturers like Medtronic, St. Jude Medical or Boston Scientific to tech start-ups like Neuralink, Kernel or Cortera, which in part work in close cooperation with several research institutes and are driven by funding from the DARPA program.

Looking forward, adaptive closed-loop stimulation systems that integrate feedback signals will ideally be able to rapidly respond to real-time patient needs and make human programming unnecessary (64). NeuroPace (California, USA) for example already provides a responsive neurostimulation system (RNS) for closed-loop cortical stimulation with FDA-approval in patients with drug-resistant epilepsy. It is capable of continuously sensing electrocorticography (ECoG) potentials (65). When recognizing a seizure-related pattern, the stimulator is activated to stop the seizure and store the ECoG potentials, date and time of seizure occurrence.

Optimally, biomarkers for adaptive closed loop DBS should be usable continuously after DBS implantation to make them applicable for clinical practice. Local field potentials and network connectivity measures based on electrophysiological signals with their high temporal resolution can already be measured with sensing DBS electrodes or other implanted neural sensors and hold great promise as biomarkers.

### Biomarkers and Control Mechanisms

Regarding closed loop adaptive DBS, a distinction has to be made between feedback signals (biomarkers) and mechanisms of control. A biomarker describes a correlative or causal relation to a clinical symptom. Adaptive control mechanisms then define how to adjust stimulation based on the evolution of biomarkers.

#### Biomarkers

##### Electrophysiological measurements

Recordings of LFPs in the basal ganglia of PD patients show oscillations at several frequencies, including oscillations at low frequencies in the delta and theta band (1~7 Hz), alpha and beta band (8–35 Hz), gamma band (35–200 Hz) and high frequency oscillations (>200 Hz). It has been demonstrated that the beta activity amplitude is correlated with motor symptom severity without medication (66–68). Moreover, it has been reported that the reduction of rigidity and bradykinesia is correlated with a decrease in beta activity (69, 70). In line with this, STN DBS and dopaminergic medication has been shown to attenuate beta activity locally (71–75), while the degree of beta activity suppression has been shown to correlate with improvement in Parkinsonian motor symptoms (71, 76). Whereas, exaggerated beta activity is associated with bradykinesia and rigidity, dyskinesia symptoms are reported to be linked to increases in low (4–8 Hz) and gamma frequencies (60–90 Hz) (77, 78), akin to oscillatory activity observed during normal movement (79–82). High frequency oscillations (HFO), which are reported to be coupled to the phase of beta oscillations, are another promising biomarker associated with Parkinsonian symptoms, such as bradykinesia, rigidity as well as tremor, even in the ON medication state (83–85). They are typically found at ~250 Hz, while not being attenuated by dopaminergic medication, but rather shifted toward higher frequencies at 350 Hz (84–86).

Early approaches using local field potentials (LFP) as feedback signals for adaptive DBS incorporated the beta frequency amplitude as a mechanism to trigger the stimulation (87) demonstrated clinical improvement of symptoms compared to standard DBS. An approach by Meidahl et al. targets potentially pathological long beta bursts sparing supposedly functionally important short-term beta bursts (88, 89). Several other oscillatory biomarkers, such as pathological cross-frequency coupling (85, 90) or pathological coherence of neural activity between cortical and subcortical structures (91) have been reported to correlate with clinical symptoms and are discussed as potential feedback signals. Despite early success, challenges have yet to be overcome. Beta power in the STN for example correlates with rigidity and bradykinesia, but not with tremor (92, 93), which is linked to field potentials at tremor frequency. PD patients for example often show heterogenous clinical symptoms, a single, one-dimensional feedback signal might be only useful to a certain degree. Body measurements using electromyography or kinematic sensors allowing for the assessment of symptom severity and behavior could be a promising additional feedback source for adaptive DBS. For instance, Cagnan and colleagues stimulated patients with essential tremor and thalamic electrodes, while recording tremor amplitude and phase with inertial sensor units. They report that the amplitude of the tremor was modulated depending on the phase relative to the tremor cycle, at which stimulation pulses were delivered (94). Most neural biomarkers like beta frequency oscillations are multifaceted and not only linked to clinical symptoms, but also modulated during normal behavior like movement or cognition (95, 96) and are associated with medication (71, 97). Although biomarkers like beta activity seem to be stable months after DBS surgery (98, 99), it is also conceivable that they evolve with disease progression, as they are correlated with symptom severity (67), which naturally increases over time in neurodegenerative diseases.

The use of electrophysiological biomarkers in aDBS is also restricted due to an often unfavorably low signal-to-noise ratio and interference with external artifacts like movement, speaking and cognition (100). Also, stimulation can lead to artifacts when sensing is done near the site of stimulation, e.g., the sensing of β-bands in the STN with e.g., Activa PC + S can be contaminated by stimulation. This may be avoided by using ECoG sensing (101). ECoG is another invasive electrophysiological biomarker which directly records electrical potentials associated with brain activity from the cortex. When using ECoG as a biomarker in aDBS the sensing strip is implanted subdurally over the primary motor cortex during the same procedure as the electrode implantation subcortically. Gamma band activity (60–90 Hz) for example is associated with dyskinesia in PD patients and can therefore be used as a feedback signal to trigger stimulation (101).

For a detailed overview of oscillatory features related to pathological and physiological states in DBS patients, see Neumann et al. (102).

##### Neurochemical sensing

Neuronal sensor devices that detect local alterations in neurotransmitter release in response to DBS have been developed. The stimulation-evoked changes that resemble physiological neurotransmitter release are associated with the therapeutic effect of DBS (103). Grahn et al. developed a device that detects changes in dopamine concentration in rodents to adapt stimulation parameters (104). Lee et al. have developed a wirelessly controlled device, WINCS Harmoni®, which can measure *in vivo* neurotransmitter concentration across multiple anatomical targets using implanted neurochemical sensors. These devices provide real-time neurochemical feedback for closed loop control (105). Until now, the method has been used in preclinical DBS studies, but it is a promising tool for a better understanding and future improvement of a clinical application of closed loop DBS.

##### External mechanistic sensors

External wearable devices, such as accelerometers or EMG sensors can be used to infer symptoms and symptom severity like rigidity, bradykinesia and gait disorders (106, 107). Studies show that the measurement of tremor with accelerometers that adjust the stimulation frequency to tremor frequency lead to a better clinical result than conventional stimulation in patients with essential tremor (52, 94). In PD, the severity of motor dysfunction can be measured with a wireless external sensor device which is integrated into a smart glove containing two touch sensors, two 3D-accelerometers and a force sensor to assess tremor, rigidity and bradykinesia of hand and arm (108). Heldman et al. devised software to automatically optimize stimulation settings based upon objective motion sensor-based motor assessments. To assess symptom severity, a motion sensor was placed on the index finger of the more affected hand. The software then guided a procedure during which stimulation on each contact was iteratively increased. This was followed by an automated assessment of tremor and bradykinesia severity. After completing assessments at each setting, a software algorithm determined stimulation settings, leading to improved tremor and bradykinesia scores by an average of 35.7% (107, 109).

#### Control Mechanisms

##### Beta threshold targeting

One of the earliest approaches to adaptive closed loop DBS was beta threshold targeting. When the amplitude of oscillatory activity in the β-band exceeds a defined threshold, stimulation is turned on. It has already been shown that this approach can improve the therapeutic effect compared to standard DBS (87). Alternatively to threshold targeting, excessive β-synchronization in PD patients may selectively be regulated via aDBS by targeting pathological long β bursts while leaving possibly functionally relevant short bursts of β activity unaffected (88). However, as described above, one problem of this approach is that not only beta oscillations but also beta oscillatory characteristics, such as burst length are not only related to symptom severity, but also to medication and behavior (75, 110).

##### Noise cancellation

Cagnan et al. suggest a tool to detect the patient's tremor with an accelerometer attached to the affected hand, as described above. Using the effect of noise cancellation, a control mechanism based on this external mechanistic sensor switches on the thalamus stimulation in specific phases of the essential tremor (52). In this work the modulation of tremor turned out to depend on the phase of stimulation relative to the tremor cycle. However, only stimulation during the first half of the tremor cycle resulted in a reduction of tremor whereas during the second half of the tremor cycle harmonics in tremor were inducted (52). Also in PD patients, the effect of noise cancellation was used to cancel cortical oscillations within the tremor network with non-invasive transcranial alternating current stimulation (tACS) which can reduce the amplitude of resting tremor by 50% (111).

##### Stimulation on demand

Measuring biomarkers in real-time can be used for stimulation on demand in aDBS. Herron et al. used cortical electrodes sensing β-band desynchronization in ET patients when a movement was started. This desynchronization then triggered the stimulation to reduce the tremor while stimulation was switched off in resting state (112). Due to a delay in stimulation initiation, tremor at the beginning of a movement could not be prevented. One way to improve this would be if one is able to predict movement before it occurs.

##### Coordinated reset stimulation

An alternative stimulation protocol is the temporal stimulation pattern coordinated reset stimulation for research application (113). Abnormal neuronal synchrony in neurological diseases can be addressed by coordinated reset stimulation that delivers brief high-frequency pulse trains through different stimulation contacts of the DBS lead to reset abnormal synchronization. In PD the basal ganglia structures STN and GPe are known to generate rhythmic synchronized oscillations which are associated with PD symptoms(114). Coordinated reset stimulation can decrease these abnormal synchronous beta oscillations and hence improve bradykinesia and rigidity (115).

## Limitations and Future Perspectives

### Moving From Invasive to Non-invasive DBS

Although the implantation of DBS electrodes is a well-established procedure in movement disorders, it comes along with surgical risks and complications. Thus, a non-invasive approach could be a future direction. Non-invasive aDBS is proposed by Grossman et al. who have developed an experimental strategy in mice to target deeply situated neurons without manipulating the overlying cortex by applying high-frequency oscillating fields in different locations outside the brain (116). The interference between two applied fields cancels out the high-frequency activity, while an oscillation of low frequency corresponding to the difference between the two frequencies can emerge. With this low frequency neurons situated deeply in the hippocampus can be activated. The suggested approach is limited by the size of human brain that is much bigger than mouse brain and hence, more difficult to target deeply located structures, and by whether neural networks in the stimulation paths remain unaffected also in a larger brain (117). Another non-invasive approach is optogenetic stimulation, which was developed over the last decade. Optogenetics can selectively activate neurons deep in the rodent brain by using light to control neuronal ion channels *in vivo*. Thus, neural circuits can be manipulated by precise excitation and inhibition of specific circuit elements, moving from invasive toward non-invasive DBS (118, 119). Currently, optogenetics still require a chronically implanted optical fiber, hence, it is not yet a completely non-invasive technique.

However, the non-invasive approaches still need to be investigated much further. So far they have only been studied in animal models.

### Future Perspectives

Most existing approaches to adaptive DBS so far have in common that they are carefully engineered based on a core principle and allow for a specific action given a certain signal. However, these approaches do not allow for learning optimal individual signal properties and control algorithms. In addition, each biomarker and control mechanism has its specific drawback as discussed above.

**Figure 1:**
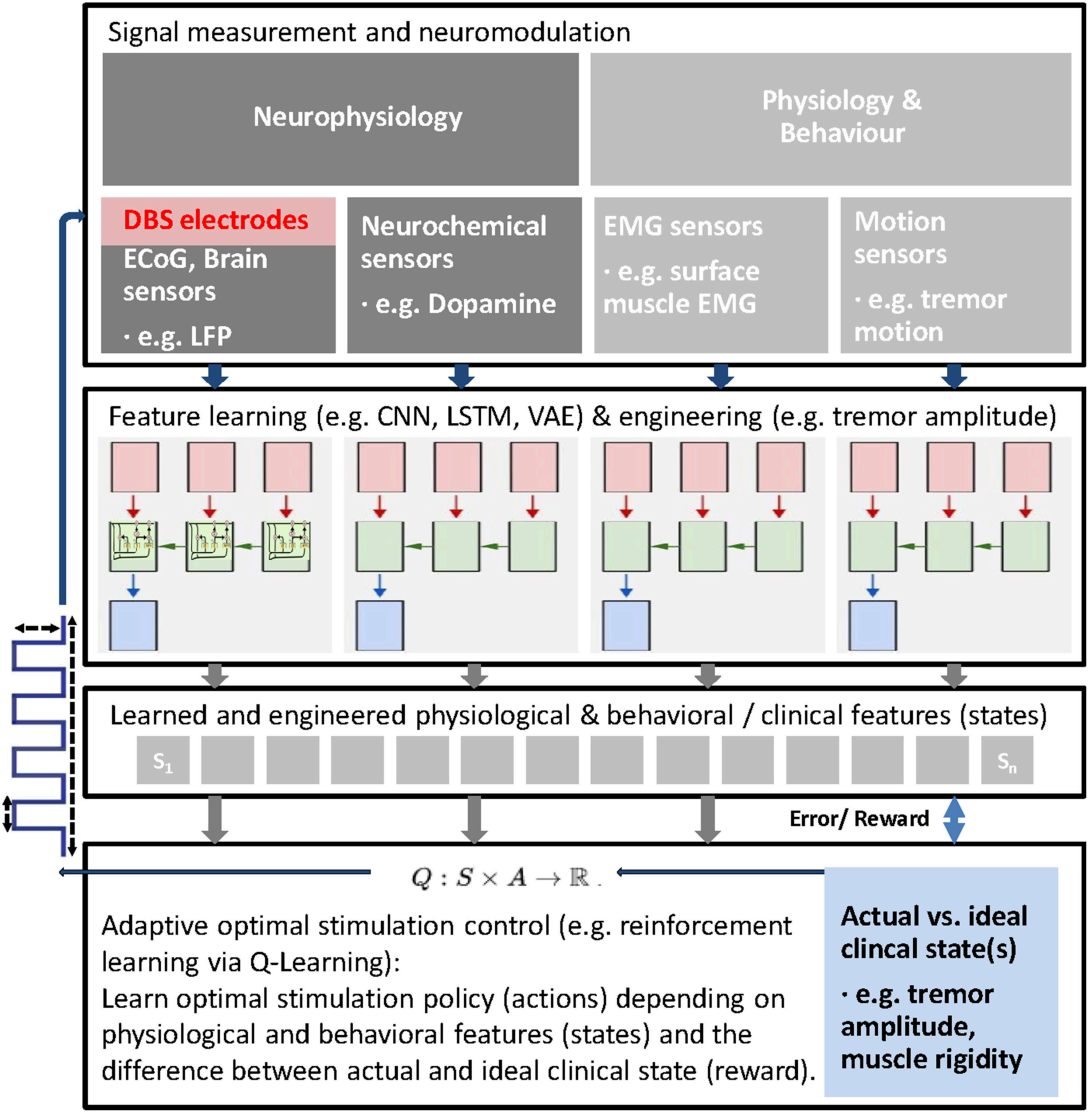
Schematic of general adaptive closed loop DBS for adaptive adjustment of deep brain stimulation (DBS) parameters based upon real time patient measurements, such as electrophysiological signals (e.g., LFP, ECoG, EMG), neurochemical parameters and behavioral measurements and machine learning. First, latent features from different possible signal sources are learned using machine learning approaches to extract behavioral (clinical) states (e.g., bradykinesia, rigidity, tremor) and corresponding and predictive latent neural states (e.g., beta and high frequency oscillations). Then, actual states are compared with ideal states to compute a reward and stimulation parameters (e.g., VTA, stimulation frequency, etc.) adjusted and finally learned via reinforcement learning (Q-Learning is shown as an example). In this closed-loop paradigm, the stimulation parameters (actions) are adjusted within clinical limits based on the reward and the extracted latent states.

As a future direction, latent features derived from different signal sources could be used in parallel to establish a feedback driven stimulation algorithm based on the analysis of behavioral and physiological data and a suitable control mechanism. By integrating parameters derived from different sources, such as kinematic and electrophysiological measurements and other sensor like electromyography, patient state and disease symptoms severity and underlying neural activity could be ultimately learned and classified end to end (102, 120–122), using machine learning algorithms (Figure 1).

In case that physiological and behavioral features, describing the neural and clinical state of the patient, can be reliably decoded and ideally predicted from measurements, reinforcement learning could be another option to learn and optimally control stimulation paradigms and optimize the clinical state (Figure 1). Reinforcement learning can provide optimal control in an environment with unknown transition probabilities (123). In reinforcement learning, an agent, in this case the DBS stimulation controller interacts with an uncertain environment, i.e., stimulating a mixture of neural structures with certain stimulation parameters with the goal to maximize a numerical long term reward, in this case the (long term) clinical improvement of the patient. Through the learned policy after training the controller ideally has identified the right stimulation action in every state (124).

A simple version of this idea could be realized in patients with tremor dominant PD. The amplitude of the tremor can be measured with kinematic sensors and then be used to describe the clinical state of the patient. Such a signal could then serve as a reward signal for reinforcement learning, with the reward simply being the difference between optimal clinical state (no tremor amplitude) and actual clinical state (actual tremor amplitude). With such an approach, the optimal stimulus could be learned and adjusted based on feedback signals, closing the loop. Alternative stimulation protocols and parameters (such as electrode contact, VTA, pulse-frequency, -width, -amplitude, -shape, timing relative to neural activity, etc.) could then be evaluated within a clinically acceptable range of stimulation energy. However, the vast amount of free parameters in DBS programming introduces a potentially very large search space to evaluate during reinforcement learning, even when constraining the search space to clinically acceptable parameters. Algorithms for reinforcement learning are commonly either model-free or model-based. While in model-free learning, the agent simply relies on trial-and-error experience to learn a policy that optimizes immediate and future reward, in model-based learning, the agent exploits previously learned lessons (125). Although model-free deep reinforcement learning algorithms are suited for learning a wide range of applications, they often require millions of training iterations to achieve good performance (126, 127), rendering this approach inappropriate for adaptive DBS trials in humans. In model-based reinforcement learning, experience is used to construct a model of the world, describing the transitions between states and associated outcomes, while suitable actions are chosen by searching or planning in this world model (128). To learn such models in the first place, however, a large number of training trials would also likely be required. Possibly animal models could help pioneering such an approach (129). Ultimately, only interventional studies can prove causal relationships and in this case the effects of adaptive deep brain stimulation on the clinical and overall state of the patient. However, applying countless experimental perturbations, which are necessary to gather enough observational data to learn from, can be costly and time consuming, even when done in animal models. Inferring the causal structure of brain networks from neuroimaging data is an important goal in neuroscience (130, 131) and various methods, such as Granger causality (132, 133), dynamic causal modeling (134, 135), structural equation modeling (136, 137) and causal Bayesian networks (138, 139) have been developed to infer causal relations from brain imaging data. Recently, van Wijk et al. applied dynamic causal modeling to explore the cortical-basal ganglia-thalamus loop in patients with PD and to study pathways that contribute to the suppression of beta oscillations induced by dopaminergic medication (140). Also recently, Bogacz et al. described a coupled oscillator model to predict the effects of deep brain stimulation (141). Ideally, causal inference methods based on i.e., causal Bayesian networks could also help give testable predictions on the effects of external manipulations (142), such as the effects of deep brain stimulation. In this way, different adaptive approaches could be explored or learned *in silico* and the number of interventional studies, that are required to establish an approach, could be reduced substantially (143).

## Searching Strategy

This review is based on expert opinions and does not follow a systematic searching strategy.

## Author Contributions

FH, JM, TK, and KB conceived the project. FH and CP did the literature search and wrote the manuscript. TK, JM, and KB wrote the manuscript.

### Conflict of Interest Statement

The authors declare that the research was conducted in the absence of any commercial or financial relationships that could be construed as a potential conflict of interest.
